# RETRACTED: Abdel-Shafi et al. Isolation and Characterization of Antibacterial Conglutinins from Lupine Seeds. *Molecules* 2023, *28*, 35

**DOI:** 10.3390/molecules30051106

**Published:** 2025-02-28

**Authors:** Seham Abdel-Shafi, Mona El-Nemr, Gamal Enan, Ali Osman, Basel Sitohy, Mahmoud Sitohy

**Affiliations:** 1 Botany and Microbiology Department, Faculty of Science, Zagazig University, Zagazig 44519, Egypt; hegazyseham@yahoo.com (S.A.-S.); mohamednoor220@yahoo.com (M.E.-N.); gamalenan@ymail.com (G.E.); 2 Biochemistry Department, Faculty of Agriculture, Zagazig University, Zagazig 44511, Egypt; aokhalil@zu.edu.eg; 3 Department of Clinical Microbiology, Infection and Immunology, Umeå University, SE-90185 Umeå, Sweden; 4 Department of Radiation Sciences, Oncology, Umeå University, SE-90185 Umeå, Sweden

Following publication, concerns were brought to the attention of the Editorial Office regarding image inconsistencies within this study [1].

Adhering to our complaints procedure, an investigation was conducted by the Editorial Office and the Editorial Board which confirmed the presence of repeated visual features within Figure 1. While the authors collaborated with the Editorial Office during the investigation, they were unable to satisfactorily explain the overlapping visual features, and the Editorial Board could not confirm the validity of the images from the raw material provided. As a result, the Editorial Board has lost confidence in the validity of the findings and decided to retract this publication [1], as per MDPI’s retraction policy (https://www.mdpi.com/ethics#_bookmark30).

This retraction was approved by the Editor-in-Chief of the *Molecules* journal.

The authors did not agree to this retraction.
